# Mediation effects of basic psychological needs and motivation among coach leadership style on the subjective well-being of adapted sports athletes’ members of the special olympics

**DOI:** 10.1371/journal.pone.0298297

**Published:** 2024-04-15

**Authors:** Ana Domingues, Jorge Santos, Marco Batista, João Serrano, Samuel Honório, João Petrica

**Affiliations:** 1 Polytechnic Institute of Castelo Branco, Castelo Branco, Portugal; 2 SHERU–Sports, Health and Exercise Research Unit–Polytechnic Institute of Castelo Branco, Castelo Branco, Portugal; Polytechnic Institute of Coimbra: Instituto Politecnico de Coimbra, PORTUGAL

## Abstract

**Introduction:**

Subjective well-being has numerous indicators of global well-being, however, the most important are life satisfaction and attachments, which can be both positive and negative. The leadership of the coach is an integral part of the process that cares for the relationship of the athlete and coach, where the coach has a fundamental role on the athlete, and consequently on his subjective well-being.

**Objective:**

The study has the purpose to analyse if and in what way the Basic Psychological Needs—relationship, autonomy and social relationship (mediator 1, M1) and the autonomous motivation (mediator 2, M2) mediate the relationship of the variables of democratic style (independent variable, X) with subjective well-being–life satisfaction and positive attachment (dependent variable, Y).

**Methods:**

In this cross-sectional study, participated 94 athletes with Intellectual and Developmental Difficulty (IDD), aged between 11 and 63 years (ẋ = 32.6 ± SD = 13.8 years) of both genders. Statistical analysis was performed using multiple serial mediation models, using the macro PROCESS *for* SPSS, version 3 (model 6), using the *bootstrap method* with 10000 samples.

**Results and Conclusions:**

There is a mediation effect between those of basic psychological needs and autonomous motivation, exercised between the democratic leadership profile of the coach and the subjective welfare of the athlete. On the other hand, autocratic style has a significant direct effect on the increase in basic psychological needs and controlled motivation. However, the democratic style is more consistent in this type of analysis. Our results reinforce the importance of promoting a democratic leadership style on the part of the coach in their athletes, enhancing a direct effect on life satisfaction and positive attachment in athletes adapted with IDD members of the Special Olympics.

## Introduction

Nowadays the regular practice of Physical Activity (PA) is already recognized as an enhancer of a healthier lifestyle, where its practice contributes to the development of physical [1], psychological [2], and social [3]. The reality proves that the sports participation of people with intellectual disabilities has a very positive impact on physical condition, self-esteem, employability, development of motor, cognitive and socialization skills and also, the practice of PA also promotes opportunities for social integration [4].

This study investigates athletes with IDD. The definition of IDD seems to have been accepted, playing with the evaluation of intellectual and adaptive criteria, whose limitations, two below-average standard deviations, are expressed in the conceptual, practical, and social competencies before the age of 18 [5], during the developmental period [6]. The key word of this definition, and contrary to what can be immediately pointed out are not the limitations, but its contextual expression, with repercussions on the (in)visibility of the person with IDD [7]. Recent investigations [8–14]. reveal the importance of analysing the act of teaching and especially the study of student thinking. Learning any motor skills requires the selection of information that may be contained in the environment and/or provided by the teacher or technician. The coach’s role is to optimize the potential of the team or the sportsman, which requires constant decision-making on a wide variety of aspects and factors [15]. The training of the coach, the leadership style he exercises and the way he plans the training process are absolutely important and fundamental variables in the coach-sports relationship and may enhance or limit the continuity of the sports participation of these two agents [16]. Sport is an activity that obeys a structure perfectly defined by rules and technical and tactical dimensions, where coaches play a key role in sportsmen, since they are considered by them as leaders and experts [16]. We noticed [17] that the Adapted Sports coach who works with athletes with IDD opts for a more democratic decision style.

Subjective well-being is the degree to which an individual judges the overall quality of his life in a favourable way. For this, the individual uses two components: one affective and the other cognitive. Being the affective that refers to their emotional reactions (being emotions, feelings and moods) that can be described as positive or negative; and also, cognitive concerns life satisfaction or cognitive assessment of life circumstances [18]. The same author points out that the idea that underlies the concept of subjective well-being is that human beings, continuously, make assessments about their life, which generate emotional reactions which can be pleasurable or distant.

There are some studies on the subject, however, more research continues to be needed to clarify the relationship of the coach’s leadership style in the subjective well-being of the athlete with IDD, specifically. Our objectives were: i) to analyse whether and how the basic psychological needs and the motivation autonomous mediate the relationship of the variables of the democratic leadership style, and also the motivation controlled with the autocratic style, with the subjective well-being of athletes with IDD. According to the existing literature, we hope to find associations between the democratic leadership style of the coach and the subjective well-being of the athlete with IDD, in the relationship of satisfaction with the athlete’s life, of his autonomous motivation and in the basic psychological needs–autonomy, competence and social relationship of the athlete.

Mediation and moderation analyses are used to establish evidence or test hypotheses about the mechanisms that explain how certain effects happen or under what conditions they facilitate or inhibit in these effects [19]. In our study, we are in the presence of multiple mediation, as they involve two mediating variables.

This investigation intends to analyse if and in what way the Basic Psychological Needs of relationship, autonomy and social relationship and the autonomous motivation mediate the relationship of the variables of democratic style with subjective well-being, life satisfaction and positive attachment.

According to this main objective, the following research questions were established:

- Which are the levels of self-determination and subjective well-being of athletes participating in the Special Olympics of Portugal?
- Which are the levels of self-determination and subjective well-being of athletes according to their gender?
  ○ Which are the levels of self-determination, subjective well-being of athletes and the influence of the democratic and autocratic style of their coaches?
- Which are and how they are associated the variables of basic psychological needs (autonomy, competence and social relationship), motivation (autonomous motivation, controlled motivation and amotivation), satisfaction with life, positive and negative affects, democratic and autocratic styles?
- How the Basic Psychological Needs–relationship, autonomy and social relationship, and autonomous motivation and controlled motivation mediate the relationship between the variables of the democratic style and the autocratic style with subjective well-being–satisfaction with life and positive and negative affections?

## Materials and methods

### Study design

This study is of quantitative nature of transversal cut, because the researcher defines the variables operationally, collects neatly verifiable data from the participants and analyses them with the help of statistical techniques [20]. The study was approved by the Ethics Committee Group of the Department of Sports and Well-being of the Polytechnic University of Castelo Branco, under the number 234/2022.

Sample

Participants were selected for convenience. A total of 94 participants aged between 11 and 63 years (ẋ = 32.6 ± SD = 13.8), of which 71.3% were male and 28.7% female, with a different number of hours of weekly practice, up to two hours of training (36.2%), 3 to 5 hours of training (39.4%), 6 to 8 hours of training (19.1%) and 9 hours or more of training (5.3%), with ẋ = 5.23 ± SD = 4.60 years of practice, competitors of various individual sports (42.6%), collective modalities (16%), and individual and collective modalities (41.5%). Only athletes with IDD were used for data analysis. Participants were recruited from institutions/schools with aggregation to the SOP (Special Olympics Portugal). Only participants were able to answer the questionnaires, even if, with the support of a significant one. All participants were informed about the objectives of the study and gave their written informed consent informed consent to participate in the study. The participants evaluated were those who competed specifically in the 2012 special edition of Special Olympics Portugal.

### Instruments and procedures

The Portuguese versions of the scales described below have been used for data collection. The application of the scales was made in the presence of a significant one for the athlete, given his condition of intellectual and developmental difficulty.

### Basic psychological needs exercise scale (BPNES)

The theory of Basic Psychological Needs is considered and aims to be a pre-requirement for the ideal functioning of the organism’s integrative processes, referring to the three basic psychological needs (autonomy, competence and social relationship), which when satisfied promote good development and maintenance psychological health and/or personal well-being [14]. The Portuguese version of the BPNES questionnaire, validated for the sport context in Portuguese [21], and developed specifically for the exercise field, was used to collect data regarding Basic Psychological Needs. The questionnaire has a scale that consists of 12 items distributed across 3 dimensions that reflect the basic psychological needs of the self-determination theory: perception of autonomy, perception of competence and perception of social relationships, with each dimension consisting of 4 items that can be classified considering a Likert-type scale with 5 response levels. This instrument had a total Cronbach’s Alpha of 0,681.

### Behaviour regulation sport questionnaire (BRSQ)

The BRSQ [22] consists of 24 items, divided into 6 subscales evaluated according to a Likert-type scale with 7 response alternatives. These items reflect the types of motivation underlying the SDT motivational continuum [14], namely amotivation, controlled motivation (external motivation, introjected motivation) and autonomous motivation (identified motivation, integrated motivation and intrinsic motivation). This scale is validated for the sporting context in Portuguese [14]. Motivation arises when an individual expresses interest in achieving a certain objective. Thus they state that “motivation is the force that compels an event to happen” [31], it presents the existence of intrinsic motivation, identified regulation, introjected regulation, external regulation and demotivation on a continuum of self-determination. This instrument had a total Cronbach’s Alpha of 0,735.

### Satisfaction with life scale (SWLS)

The Life Satisfaction Scale, as the name suggests, was used to assess life satisfaction as a cognitive construct [23]. This scale consists of 5 items and consists of indicating, using a 7-point Likert scale, the degree of satisfaction according to each item. In relation to the validation process of the Portuguese version of the Satisfaction with Life Scale [54], through confirmatory factor analysis, the goodness-of-fit indices were considered adequate. This instrument had a total Cronbach’s Alpha of 0,842.

### Positive and negative affect schedule (PANAS)

The Positive and Negative Affects Scale, originally translated into Portuguese [24], with the aim of evaluating subjective well-being and affectivity, is composed of twenty items, as a way of evaluating positive affects, through ten adjectives, and also negative affects, to which the remaining adjectives correspond. Each item on this scale is assigned a value corresponding to a Likert scale that varies between 1 and 5. This scale was validated for the Portuguese language [24] and validated in Portuguese under the name Sports Leadership Scale (SLS). It is a scale that assesses the coach’s perception of his own behavior (self-perception version), the athletes’ perception of their coaches’ leadership behaviors (perception version), as well as the athletes’ preference for the coach’s behavior (preferences version). It also analyzes behaviors in five different dimensions: training, instruction behavior, social support behavior, reinforcement behavior, democratic behavior and autocratic behavior. The ELD is presented in the form of 40 questions for each version and in each of the questions, the athlete or coach could choose one of five possible answers, given on a Likert-type scale; (1 = Never; 2 = Rarely; 3 = Occasionally; 4 = Often; 5 = Always). This study only used the perception version that athletes have regarding their coach. However, taking into account the population to which this instrument will be applied, we chose to use a pictographic scale so that the respondent’s response is clearer and more perceptible. This instrument had a total Cronbach’s Alpha of 0,763.

### Leadership scale for sport (LSS)

The LSS, validated in Portuguese [25], is a scale that assesses the coach’s perception of their own behavior (self-perception version), the perception that athletes have about the leadership behaviors of their respective coaches (perception version), as well as such as the athletes’ preference for the coach’s behavior (preferences version). This study used only the perception version. It also analyzes behaviors in five different dimensions: training, instruction behavior, social support behavior, reinforcement behavior, democratic behavior and autocratic behavior. The LSS is presented in the form of 40 questions, and in each of the questions, the athlete could choose one of five possible answers, given on a Likert scale, which varies between 1 and 5. However, taking into account the population in which this instrument will be applied, we chose to use a pictographic scale so that the respondent’s response is clearer and more perceptible. This instrument had a total Cronbach’s Alpha of 0,592.

In Table 1 we can see a general characterization of the results, in relation to the variables evaluated, where the minimum, maximum, mean values, standard deviation, Cronbach’s alpha values for each category and also the respective Kolmogorov Smirnov normality test values are presented. When analyzing the results of the Kolmogorov Smirnov test, we can see that only the positive affects variable (0.070) assumes a normal distribution of data (Sig.>0.05). On the other hand, the remaining variables assumed non-normal distribution. Cronbach’s Alphas of variables with a value greater than and equal to 0.80 are considered “moderate to high”, while variables with values between 0.70 and 0.60 are considered to have a “low level”. This values allow us to guarantee that the questionnaire obtained appropriate reliability. Satisfaction with life and the dimension of negative affects were the variables that achieved the highest reliability index, presenting an alpha value of 0.84, followed by autonomous motivation with 0.81, controlled motivation and the dimension of the relationship (Needs Basic Psychological Needs) with 0.80, the competence dimension (Basic Psychological Needs) and positive affects with 0.70 and finally the amotivation and the autonomy dimension (Basic Psychological Needs) with 0.62 and 0.60, respectively. Variables with a value greater than and equal to 0.80 are considered “moderate to high”, while variables with values between 0.70 and 0.60 are considered “low level” [46].

**Table 1:** Descriptive statistics and normality of the motivation variables, basic psychological needs, life satisfaction, positive and negative affects of athletes with IDD participating in the SOP.

Variables	Cronbach’s alpha	KS	Mean	Minimum	Maximum	SD
**Autonomy **	0.581	0.000*	4.43	2.00	5.00	0.73
**Competence **	0.689	0.000*	4.77	3.67	5.00	0.36
**Relation**	0.774	0.000*	4.84	3.67	5.00	0.35
**Autonomous Mot.**	0.811	0.000*	6.70	4.36	7.00	0.51
**Controlled Mot.**	0.780	0.001*	2.04	1.00	6.00	1.23
**Amotivation **	0.616	0.000*	1.90	1.00	6.00	1.20
**Life satisfaction**	0.842	0.000*	6.34	1.75	7.00	1.06
**Positive affects **	0.688	0.070	4.30	2.67	5.00	0.60
**Negative affects**	0.838	0.027*	1.90	1.00	4.00	0.80

* p ≤ 0.05 does not respect the assumption of normality

In Table 2 we can see a general characterization of the results, in relation to the variables evaluated, where the minimum, maximum, mean values, standard deviation, Cronbach’s alpha values for each category and the respective Kolmogorov Smirnov normality test values are presented. The Kolmogorov-Smirnov test is a non-parametric test that allows you to determine whether a given sample can be considered as coming from a certain distribution. When analyzing the test results, we can see that only the positive affects variable (0.07) assumes a normal distribution of data (Sig.>0.05). On the other hand, the remaining variables assumed non-normal distribution. The Cronbach’s Alphas of variables with a value greater than and equal to 0.80 are considered “moderate to high”, while variables with values between 0.70 and 0.60 are considered “acceptable”, thus the values allow us to ensure that the questionnaire has achieved appropriate reliability. Fidelity reveals the precision of an instrument, that is, the consistency and stability of the measurements. Satisfaction with life and the dimension of negative affects were the variables that achieved the highest reliability index, presenting an alpha value of 0.84, followed by autonomous motivation with 0.81, controlled motivation and the relationship dimension with 0.80, the competence dimension (Basic Psychological Needs) and positive affects with 0.70, the democratic style with 0.63, amotivation with 0.62, and finally the autonomy dimension and the autocratic style with 0.60 and 0.55, respectively. Variables with a value greater than and equal to 0.80 are considered “moderate to high”, while variables with values above 0.60, which are not ideal, are acceptable [46].

**Table 2:** Descriptive statistics and reliability analysis of the variables of motivation, basic psychological needs, satisfaction with life, positive and negative affects and democratic or autocratic leadership style.

Variables	Cronbach’s alpha	KS	Mean	Minimum	Maximum	SD
**Autonomy **	0.581	0.000*	4.43	2.00	5.00	0.73
**Competence **	0.689	0.000*	4.77	3.67	5.00	0.36
**Relation**	0.774	0.000*	4.84	3.67	5.00	0.35
**Autonomous Mot.**	0.811	0.000*	6.70	4.36	7.00	0.51
**Controlled Mot.**	0.780	0.001*	2.04	1.00	6.00	1.23
**Amotivation **	0.616	0.000*	1.90	1.00	6.00	1.20
**Life satisfaction**	0.842	0.000*	6.34	1.75	7.00	1.06
**Positive affects **	0.688	0.070	4.30	2.67	5.00	0.60
**Negative affects**	0.838	0.027*	1.90	1.00	4.00	0.80
**Democratic style**	0.636	0.001*	3.47	2.50	4.25	0.40
**Autocratic style**	0.549	0.000*	1.78	1.00	3.00	0.84

* p ≤ 0.05 does not respect the assumption of normality

### Data analysis

The statistical processing of the data was performed using the *computer program Statistical Package for a Social Sciences* (SPSS) version 21.0 (IBM, Chicago, Illinois, U.S.A.).

To analyse whether and how basic psychological needs–social relationship, autonomy and competence (mediator 1, M1) and autonomous motivation and controlled motivation (mediator 2, M2) mediate the relationship between the variables of the democratic and autocratic leadership style (X) with subjective well-being–satisfaction with life, positive and negative attachment (Y). After the serialization of the data, we tested the multiple serial mediation analysis for the variables in question, in which the macro PROCESS for SPSS was used, v. 3.4.1 [26]. Mediation is a special case of indirect effect in which the relationship between the independent and dependent variable is required to be present initially [27]. The variable of the democratic style, included in the mediation, is the one that in the previous analysis (correlation and regression) had been consistently associated with satisfaction with life. The confidence intervals of *bootstrap analysis* for indirect effects are repeatedly calculated on 10,000 *bootstrap samples*, estimating the model in each of these samples, calculating indirect effects and deriving the final Confidence Interval (CI). The confidence level for all CI is 95%. An indirect effect is nonzero with a 95%CI, if the value zero is not included in the CI. If the 95% CI contains this value, the indirect effect is not statistically nonzero.

In the statistical analysis, the following levels of statistical significance *were considered α≤0*.01** and α≤0.05*for a confidence interval of at least 95%.

## Results and discussion

Multiple serial mediation analysis includes on the coach’s democratic leadership style because previous analyses (correlation and regression) show that this leadership style is most associated with life satisfaction and positive attachment. Thus, in this analysis we explore the relationship of the democratic style (X) with satisfaction with life and, later with positive affections (Y), using as mediators of this connection, the basic psychological needs–relationship, autonomy and competence (M1) and autonomous motivation (M2).

**Fig 1:**
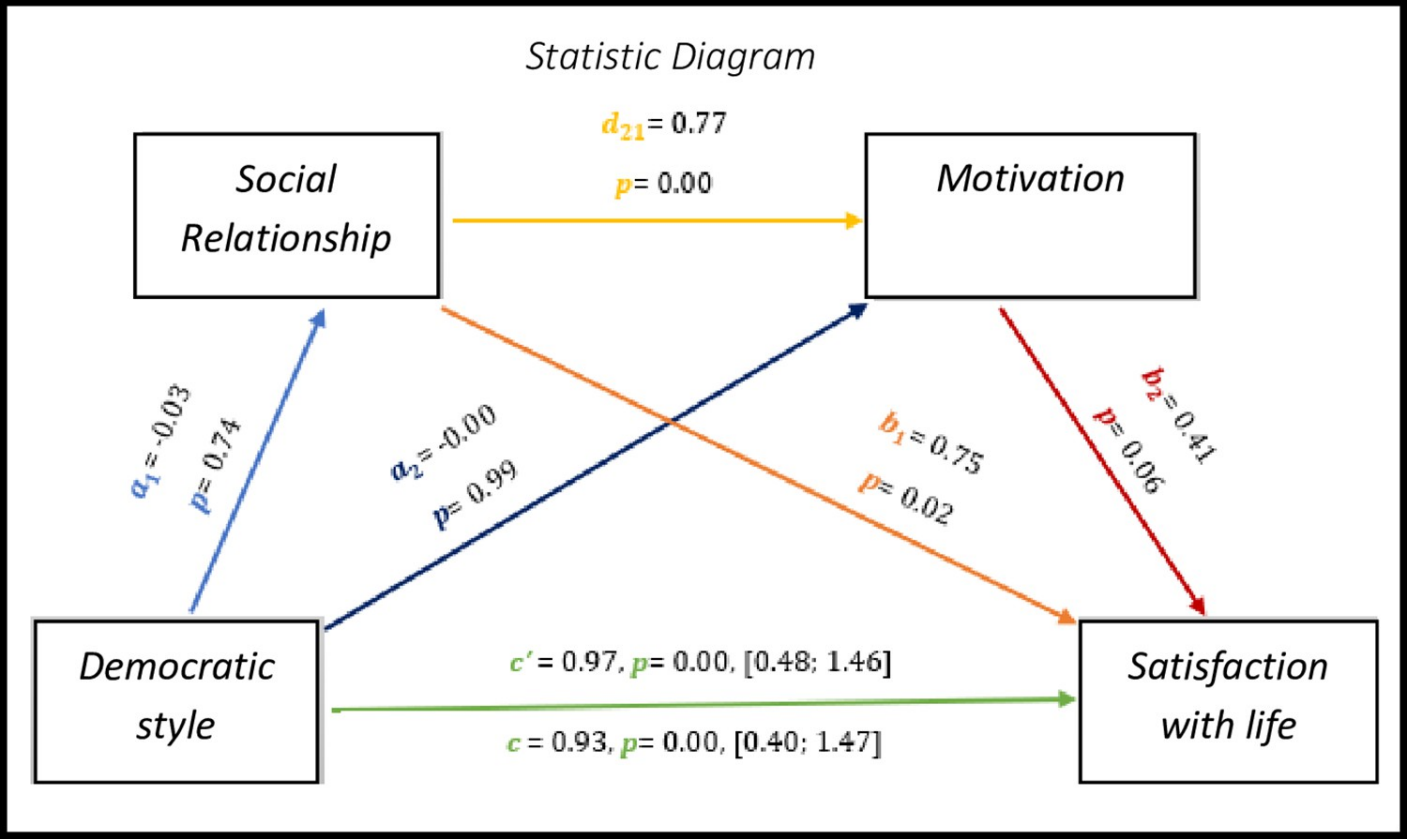
Statistical diagram of the multiple serial model for the relationship between democratic style (X), satisfaction with life (Y), social relationship (M1) and autonomous motivation (M2).

Fig *1* presents data related to the mediation process between democratic style (X), satisfaction with life (Y), relationship (M1) and autonomous motivation (M2). To explain the links between the variables, values were found for regression analyses, the first regression analysis to which the variable X is linked to M1, characterized by the coefficient; the second regression analysis connects X with M2, characterized by the coefficient, the link between M1 and M2, presented by the coefficient. Regarding the analysis of the third regression, this is the link between Y and M1, M2 and X, characterized by coefficient, and *a*_1_*a*_2_*d*_21_*b*_1_*b*_2_c′. The total effect of our mediation is presented by the coefficient *c*. The link *c’* represents the direct effect of the independent variable on the dependent variable, through the mediators.

The results point to a significant direct effect and total effect (total effect—*c* = 0.93, *p* = 0.00, 95% CI [0.40; 1.47] and direct effect—*c’* = 0.97, *p* = 0.00, 95% CI [0.48; 1.46]). In this follow-up, we can observe significant results (p<0.05) in all associations except between the democratic style–relationship, democratic style–autonomous motivation and, autonomous motivation–satisfaction with life.

As for indirect effects, we analyse:

✓ 1° *a*_1_*b*_1_(ind1): X→M1→Y - β = -0.02, SE = 0.06, 95% Confidence Interval (CI) [-0.13; 0.11];
✓ 2°: *a*_2_*b*_2_(ind2): X→M2→Y - β = -0.01, SE = 0.03, 95% CI [-0.07; 0.05] e;
✓ 3°: *a*_1_*d*_21_*b*_2_(ind3): X→M1→M2→Y - β = -0.00, SE = 0.07, 95% CI [-0.09; 0.18].

Although there is no significant effect of the democratic style with the basic psychological needs of relationship and autonomous motivation, the democratic style evidences an effective relationship with the satisfaction with the lives of athletes with IDD.

**Fig 2:**
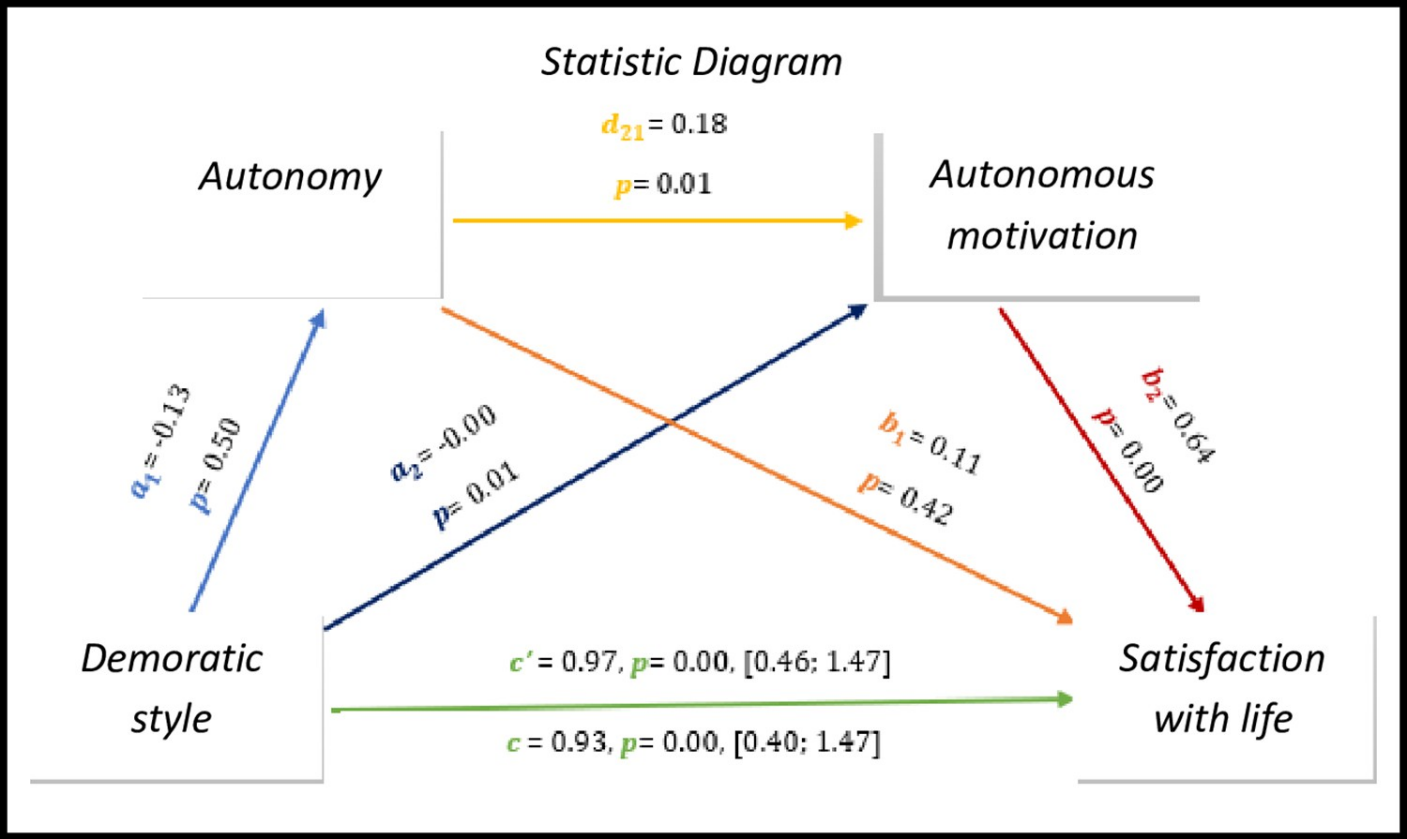
Statistical diagram of the multiple serial model for the relationship between democratic style (X), satisfaction with life (Y), autonomy (M1) and autonomous motivation (M2).

As we can see *in Fig 2,* the results show significant values with regard to direct effect and total effect, *c’* = 0.97, *p* = 0.00, 95% CI [0.46; 1.47], *c* = 0.93, *p* = 0.00, 95% CI [0.40; 1.47], respectively.

By analogy, we can observe significant results (p<0.05) in all associations except between democratic style—autonomy and autonomy- satisfaction with life (p>0.05).

As for indirect effects, we analyse:

✓ 1° *a*_1_*b*_1_(ind1): X→M1→Y - β = -0.02, SE = 0.03, 95% CI [-0.08; 0.03];
✓ 2°: *a*_2_*b*_2_(ind2): X→M2→Y - β = -0.02, SE = 0.02, 95% CI [-0.07; 0.03] e:
✓ 3°: *a*_1_*d*_21_*b*_2_(ind3): X→M1→M2→Y - β = -0.00, SE = 0.11, 95% CI [-0.20; 0.27].

Similarly, we found that the democratic style presents an effect with satisfaction with the lives of athletes with IDD, however, the same is not the case in the democratic style with the basic psychological needs of autonomy and autonomy with satisfaction with life.

**Fig 3:**
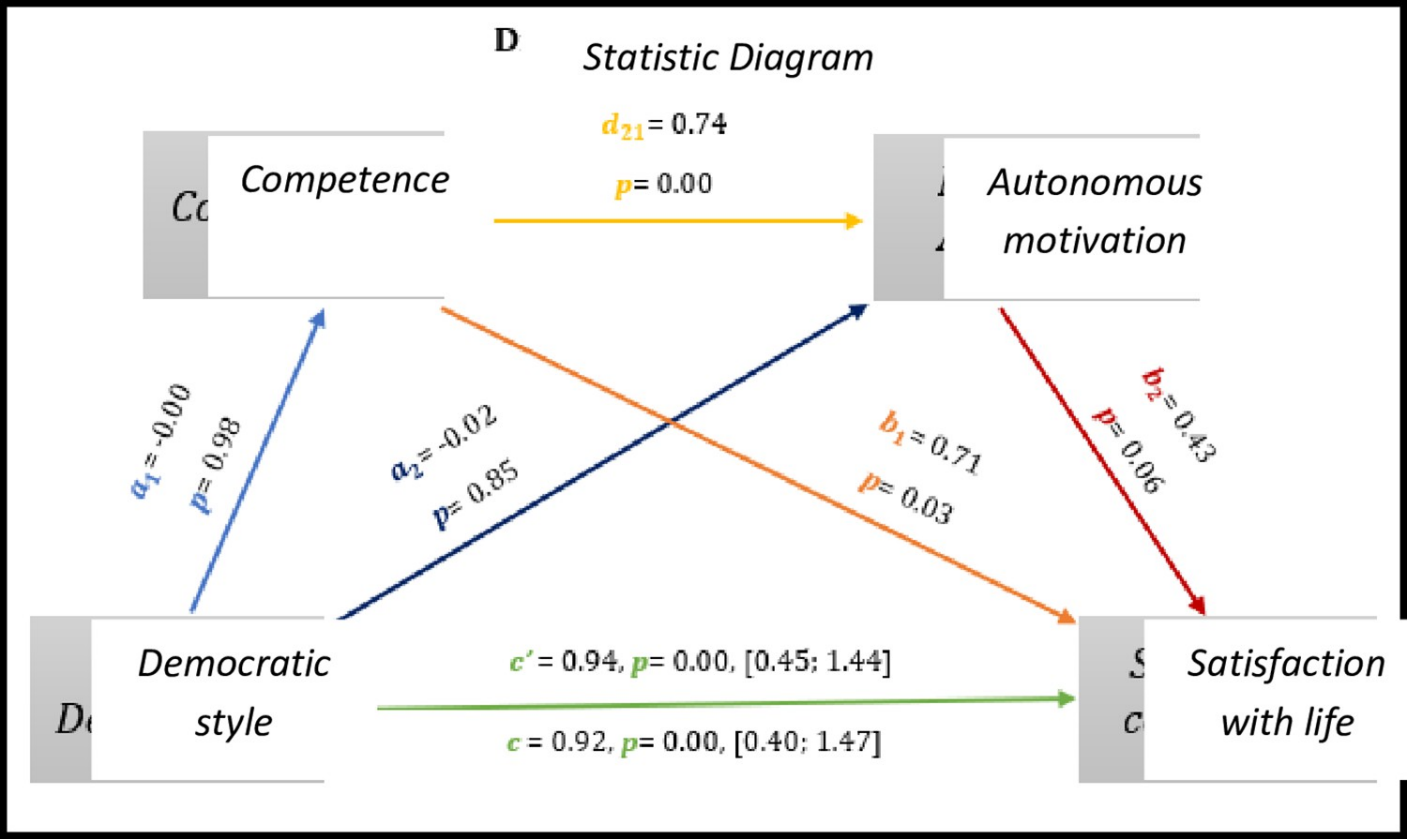
Statistical diagram of the multiple serial model for the relationship between democratic style (X), satisfaction with life (Y), competence (M1) and autonomous motivation (M2).

Based on *Fig 3,* we observed significant results regarding direct effect and total effect, *c’* = 0.94, *p* = 0.00, 95% CI [0.45; 1.44] and *c* = 0.92, *p* = 0.00, 95% CI [0.40; 1.47], respectively. Although there is no significant effect between the democratic style–competence (0.98), the democratic style–autonomous motivation (0.85) and also the autonomous motivation–satisfaction with life (0.06).

As for indirect effects, we analyse:

✓ 1° *a*_1_*b*_1_(ind1): X→M1→Y -β = -0.00, SE = 0.10, 95% CI [-0.18; 0.23];
✓ 2°: *a*_2_*b*_2_(ind2): X→M2→Y - β = -0.00, SE = 0.05, 95% CI [-0.10; 0.10] e;
✓ 3°: *a*_1_*d*_21_*b*_2_(ind3): X→M1→M2→Y - β = -0.00, SE = 0.08, 95% CI [-0.16; 0.20].

Similarly, we found that the democratic style presents a direct effect with the satisfaction with the lives of athletes with IDD.

**Fig 4:**
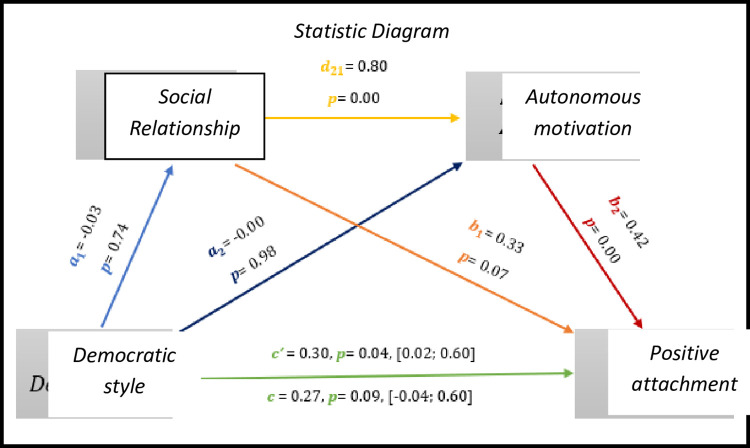
Statistical diagram of the multiple serial model for the relationship between democratic style (X), positive attachment (Y), relationship (M1) and autonomous motivation (M2).

The results of Fig 4, show that although there is no significant total effect *(c* = 0.27, *p* = 0.09, 95% CI [-0.04; 0.60]), there is a significant direct effect of the democratic style on positive attachment (*c’* = 0.30, *p* = 0.04, 95% CI [0.02; 0.60]).

As for indirect effects, we analyse:

✓ 1° *a*_1_*b*_1_(ind1): X→M1→Y - β = -0.01, SE = 0.03, 95% CI [-0.07; 0.06];
✓ 2°: *a*_2_*b*_2_(ind2): X→M2→Y - β = -0.01, SE = 0.03, 95% CI [-0.06; 0.04] e;
✓ 3°: *a*_1_*d*_21_*b*_2_(ind3): X→M1→M2→Y - β = -0.00, SE = 0.06, 95% CI [-0.09; 0.15].

Thus, we observed an association in the relationship of the democratic style to the positive affections, mediated by the variables of the relationship and the autonomous motivation.

**Fig 5:**
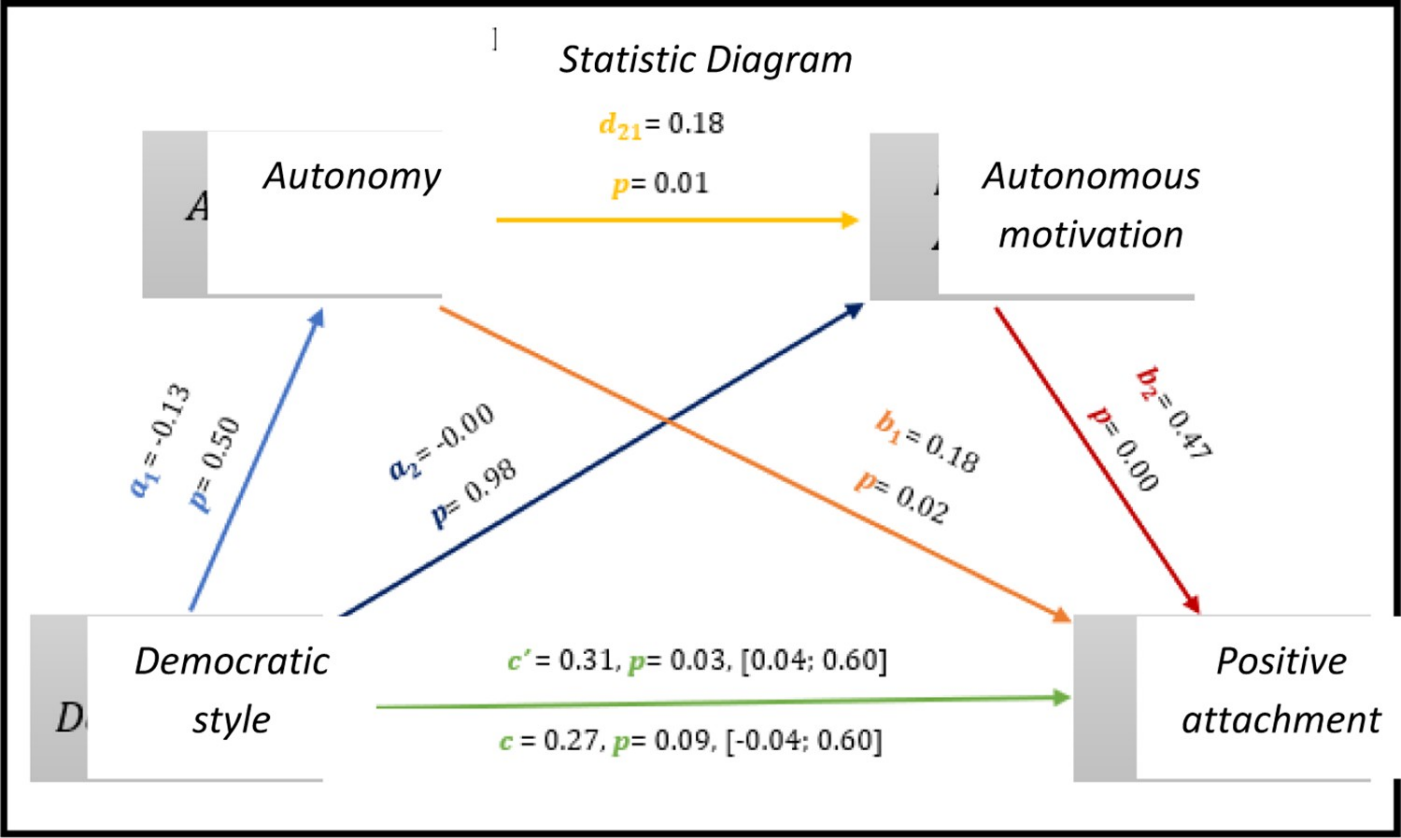
Statistical diagram of the multiple serial model for the relationship between democratic style (X), positive attachment (Y), autonomy (M1) and autonomous motivation (M2).

Through the multiple mediation analysis, in *Fig 5,* the results indicate significant values regarding the direct effect *(c’* = 0.31, *p* = 0.03, 95% CI [0.04; 0.60]). From the same point of view, we can observe significant results (p<0.05) on the part of autonomy with positive affections and autonomous motivation and also in autonomous motivation with positive effects.

As for indirect effects, we analyse:

✓ 1° *a*_1_*b*_1_(ind1): X→M1→Y - β = -0.02, SE = 0.03, 95% CI [-0.09; 0.04];
✓ 2°: *a*_2_*b*_2_(ind2): X→M2→Y - β = -0.01, SE = 0.02, 95% CI [-0.04; 0.02] e;
✓ 3°: *a*_1_*d*_21_*b*_2_(ind3): X→M1→M2→Y - β = -0.00, SE = 0.08, 95% CI [-0.14; 0.20].

**Fig 6:**
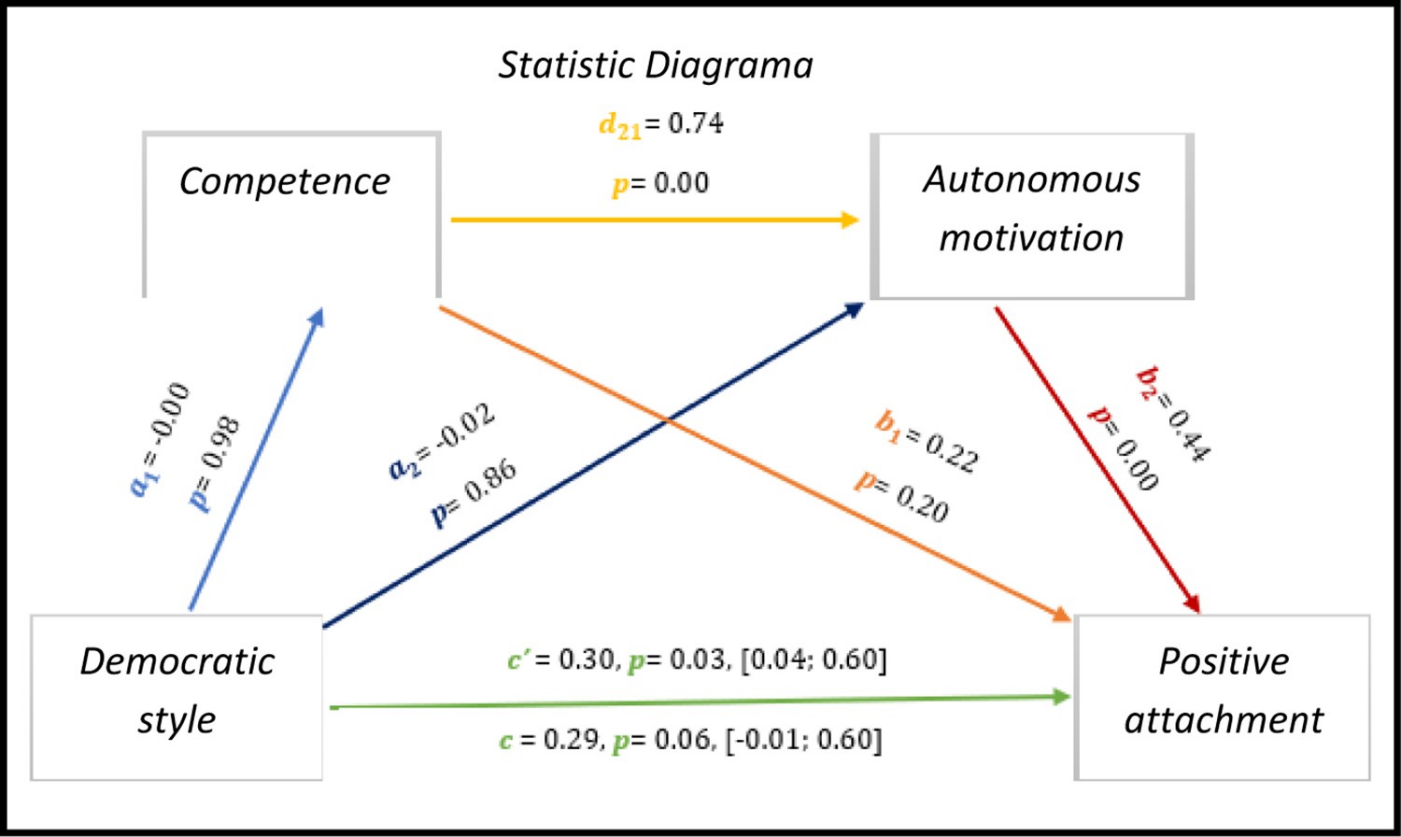
Statistical diagram of the multiple serial model for the relationship between democratic style (X), positive attachment (Y), competence (M1) and autonomous motivation (M2).

Here too we see a significant direct effect between the democratic style and the positive affections of athletes with IDD. The results of *Fig 6,* show through multiple mediation analysis significant results for the direct effect of the democratic style with positive attachment (*c’* = 0.30, *p* = 0.03, 95% CI [0.04; 0.60]), on the other hand the total effect is not significant (*c* = 0.29, *p* = 0.06, 95% CI [-0.01; 0.60]).

Although we found significant values in competence for autonomous motivation (0.00) and autonomous motivation for positive effects, all others do not present significant values.

As for indirect effects, we analyse:

✓ 1° *a*_1_*b*_1_(ind1): X→M1→Y - β = -0.00, SE = 0.04, 95% CI [-0.09; 0.06];
✓ 2°: *a*_2_*b*_2_(ind2): X→M2→Y - β = -0.00, SE = 0.04, 95% CI [-0.08; 0.09] e;
✓ 3°: *a*_1_*d*_21_*b*_2_(ind3): X→M1→M2→Y - β = -0.01, SE = 0.07, 95% CI [-0.14; 0.15].

Similarly, we see a significant direct effect between the democratic style with the positive affections of these athletes.

**Fig 7:**
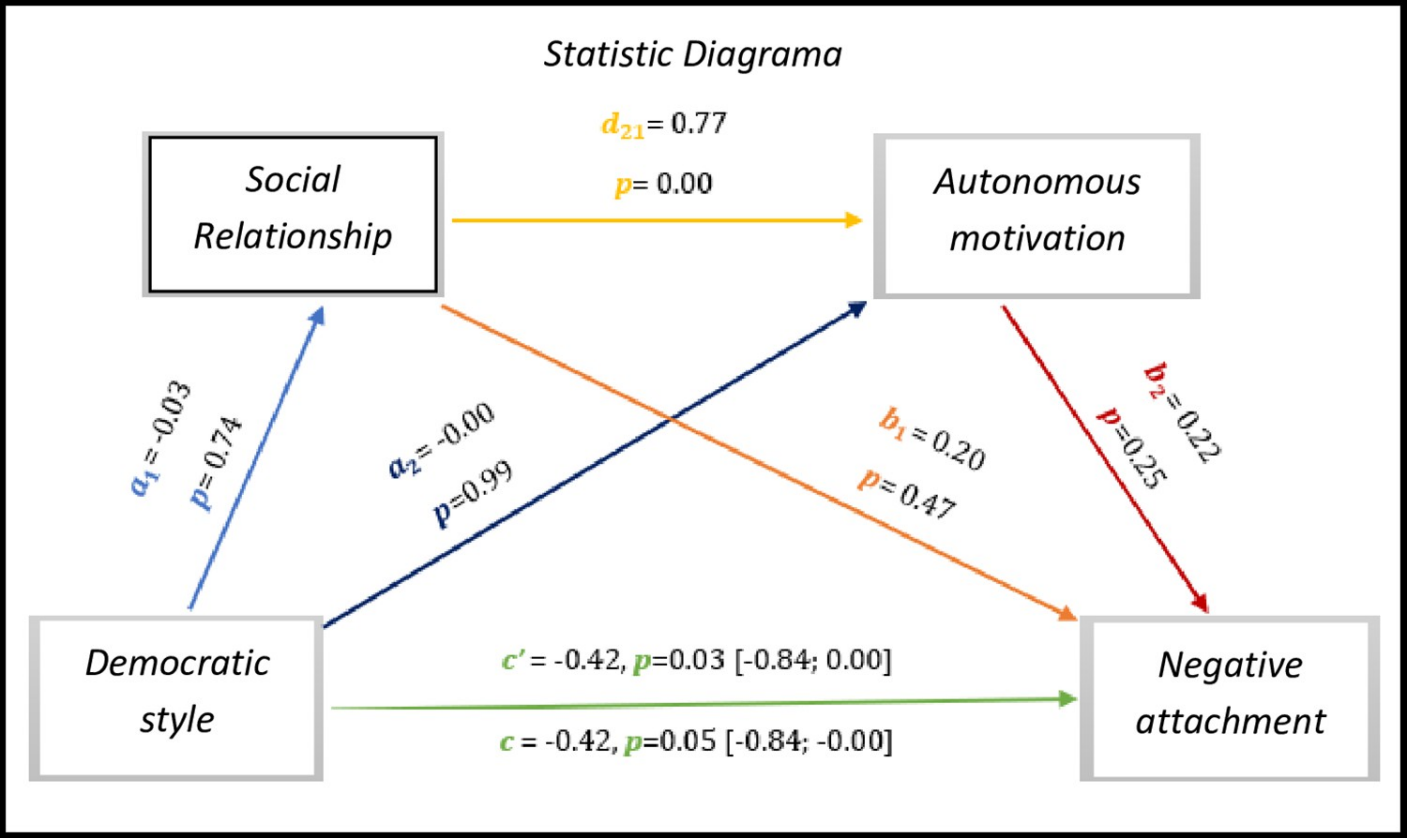
Statistical diagram of the multiple serial model for the relationship between democratic style (X), negative attachment (Y), relationship (M1) and autonomous motivation (M2).

As we can see *in Fig 7,* the results show significant values regarding the direct effect and total effect, *c’* = -0.42, *p* = 0.03, 95% CI [-0.84; 0.00], *c* = -0.42, *p* = 0.05, 95% CI [-0.84; -0.00], respectively. Although there is no significant effect between the democratic style–relationship (0.74), the democratic style–autonomous motivation (0.99), the relationship–negative attachment (0.47), and also the autonomous motivation–negative attachment (0.25).

As for indirect effects, we analyse:

✓ 1° *a*_1_*b*_1_(ind1): X→M1→Y - β = -0.01, SE = 0.02, 95% CI [-0.05; 0.04];
✓ 2°: *a*_2_*b*_2_(ind2): X→M2→Y - β = 0.00, SE = 0.04, 95% CI [-0.09; 0.07] e;
✓ 3°: *a*_1_*d*_21_*b*_2_(ind3): X→M1→M2→Y - β = 0.00, SE = 0.02, 95% CI [-0.03; 0.04].

Similarly, we found that the democratic style presents an effect with the negative attachment of athletes with IDD, however, the same is not the case in the democratic style with the basic psychological needs of relationship and with the autonomous motivation.

**Fig 8:**
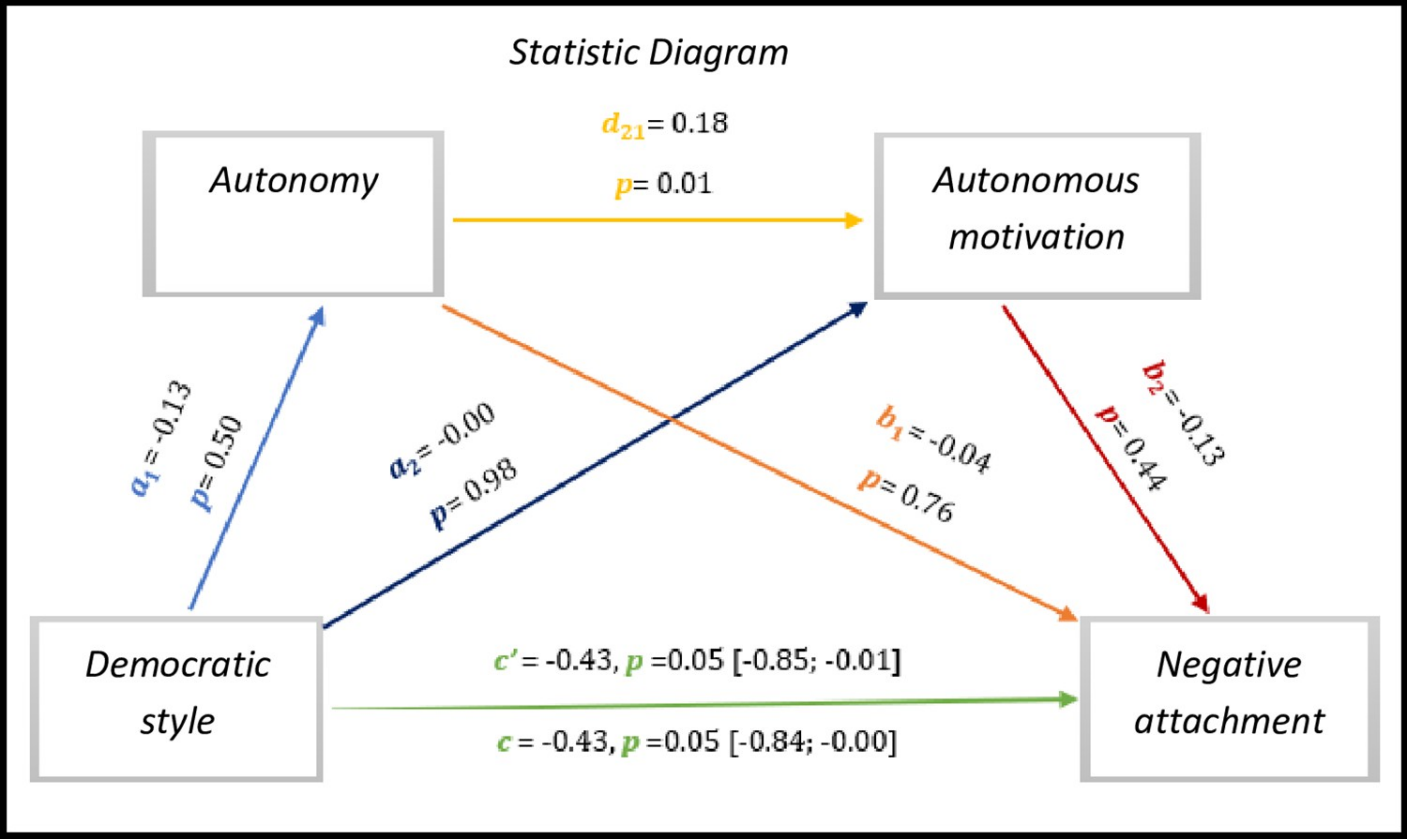
Statistical diagram of the multiple serial model for the relationship between democratic style (X), negative attachment (Y), autonomy (M1) and autonomous motivation (M2).

Based on *Fig 8,* we observed significant results regarding direct effect and total effect, *c’* = 0.43, *p* = 0.05, 95% CI [-0.85; -0.01] and *c* = -0.43, *p* = 0.05, 95% CI [-0.84; -0.00], respectively. Nevertheless, only autonomy–autonomous motivation presents a significant indirect path, which is not the case in the other connections.

As for indirect effects, we analyse:

✓ 1° *a*_1_*b*_1_(ind1): X→M1→Y - β = 0.00, SE = 0.03, 95% CI [-0.04; 0.07];
✓ 2°: *a*_2_*b*_2_(ind2): X→M2→Y - β = 0.00, SE = 0.04, 95% CI [-0.09; 0.07] e;
✓ 3°: *a*_1_*d*_21_*b*_2_(ind3): X→M1→M2→Y - β = 0.00, SE = 0.01, 95% CI [-0.01; 0.02].

Similarly, we found that the democratic style presents to have a direct effect with the negative attachment of athletes with IDD.

**Fig 9:**
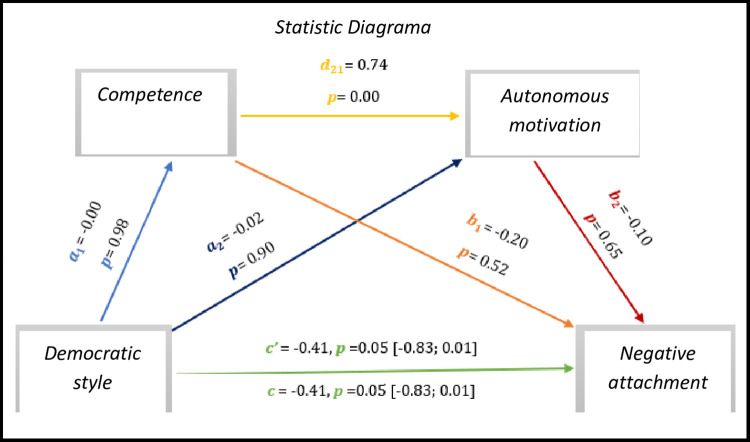
Statistical diagram of the multiple serial model for the relationship between democratic style (X), negative attachment (Y), competence (M1) and autonomous motivation (M2).

The results of *Fig 9,* we observed significant results regarding direct effect and total effect, *c’* = -0.41, *p* = 0.05, 95% CI [-0.83; 0.01] and *c* = -0.41, *p* = 0.05, 95% CI [-0.83; 0.01], respectively. Nevertheless, only competence–autonomous motivation presents a significant indirect path, which is not the case in the other connections.

As for indirect effects, we analyse:

✓ 1° *a*_1_*b*_1_(ind1): X→M1→Y - β = 0.00, SE = 0.04, 95% CI [-0.09; 0.08];
✓ 2°: *a*_2_*b*_2_(ind2): X→M2→Y - β = 0.00, SE = 0.03, 95% CI [-0.07; 0.07] e;
✓ 3°: *a*_1_*d*_21_*b*_2_(ind3): X→M1→M2→Y - β = 0.00, SE = 0.02, 95% CI [-0.05; 0.04].

Thus, we observed an association in the relationship of the democratic style to the negative attachment.

In summary, the results show that in most of all mediation models, in which life satisfaction, positive attachment and negative attachment are the *outcome*, the democratic style has a significant direct effect on the increase of basic psychological needs and autonomous motivation, even if the latter, presents higher values. In turn, basic psychological needs have a significant effect on increasing autonomous motivation.

**Fig 10:**
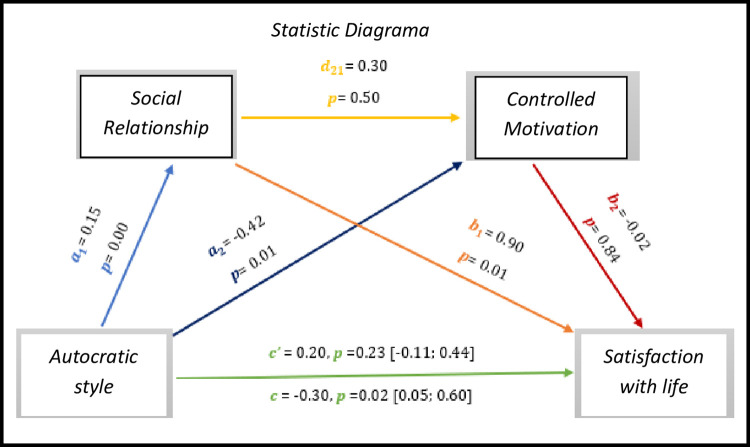
Statistical diagram of the multiple serial model for the relationship between autocratic style (X), satisfaction with life (Y), relationship (M1) and controlled motivation (M2).

Through the multiple mediation analysis, in *Fig 10,* the results indicate significant values with regard to the total effect (*c* = -0.30, *p* = 0.02, 95% CI [0.05; 0.60]), however gold side the direct effect is not significant (*c’* = 0.20, *p* = 0.23, 95% CI [-0.11; 0.44]).

As for indirect effects, we analyse:

✓ 1° *a*_1_*b*_1_(ind1): X→M1→Y - β = 0.13, SE = 0.05, 95% CI [0.04; 0.24];
✓ 2°: *a*_2_*b*_2_(ind2): X→M2→Y - β = 0.01, SE = 0.05, 95% CI [-0.08; 0.11] e;
✓ 3°: *a*_1_*d*_21_*b*_2_(ind3): X→M1→M2→Y - β = -0.00, SE = 0.01, 95% CI [-0.02; 0.01].

Although here we do not see a significant direct effect between the autocratic style and the satisfaction with the lives of athletes with IDD, some indirect and total effects are significant.

**Fig 11:**
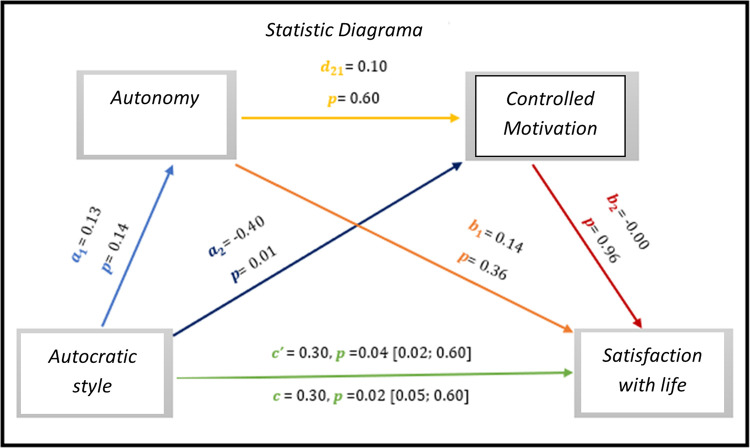
Statistical diagram of the multiple serial model for the relationship between autocratic style (X), satisfaction with life (Y), autonomy (M1) and controlled motivation (M2).

The results of *Fig 11,* show through multiple mediation analysis, significant results regarding direct effect and total effect, *c’* = 0.30, *p* = 0.04, 95% CI [0.02; 0.60] and *c* = 0.30, *p* = 0.02, 95% CI [0.05; 0.60], respectively.

As for indirect effects, we analyse:

✓ 1° *a*_1_*b*_1_(ind1): X→M1→Y - β = 0.02, SE = 0.03, 95% CI [-0.02; 0.10];
✓ 2°: *a*_2_*b*_2_(ind2): X→M2→Y - β = 0.00, SE = 0.04, 95% CI [-0.10; 0.10] e;
✓ 3°: *a*_1_*d*_21_*b*_2_(ind3): X→M1→M2→Y - β = -0.00, SE = 0.00, 95% CI [-0.01; 0.01].

Soon we see a significant direct effect between the autocratic style in the satisfaction with the lives of these athletes.

**Fig 12:**
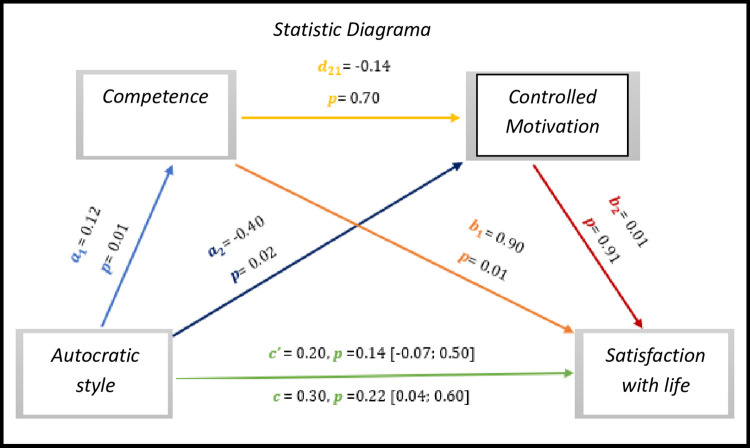
Statistical diagram of the multiple serial model for the relationship between autocratic style (X), satisfaction with life (Y), competence (M1) and controlled motivation (M2).

The results of the analysis, *in Fig 12,* show that the results indicate non-significant values, either in the direct effect (*c’* = 0.20, *p* = 0.14, 95% CI [-0.07; 0.50]), or in the total effect (*c* = 0.30, *p* = 0.22, 95% CI [0.04; 0.60]).

As for indirect effects, we analyse:

✓ 1°*a*_1_*b*_1_(ind1): X→M1→Y - β = 0.11, SE = 0.05, 95% CI [0.02; 0.21];
✓ 2°: *a*_2_*b*_2_(ind2): X→M2→Y - β = -0.00, SE = 0.04, 95% CI [-0.08; 0.08] e;
✓ 3°: *a*_1_*d*_21_*b*_2_(ind3): X→M1→M2→Y - β = -0.00, SE = 0.00, 95% CI [-0.01; 0.01].

However, the autocratic style has no effect on the satisfaction with the lives of the athletes in our study.

**Fig 13:**
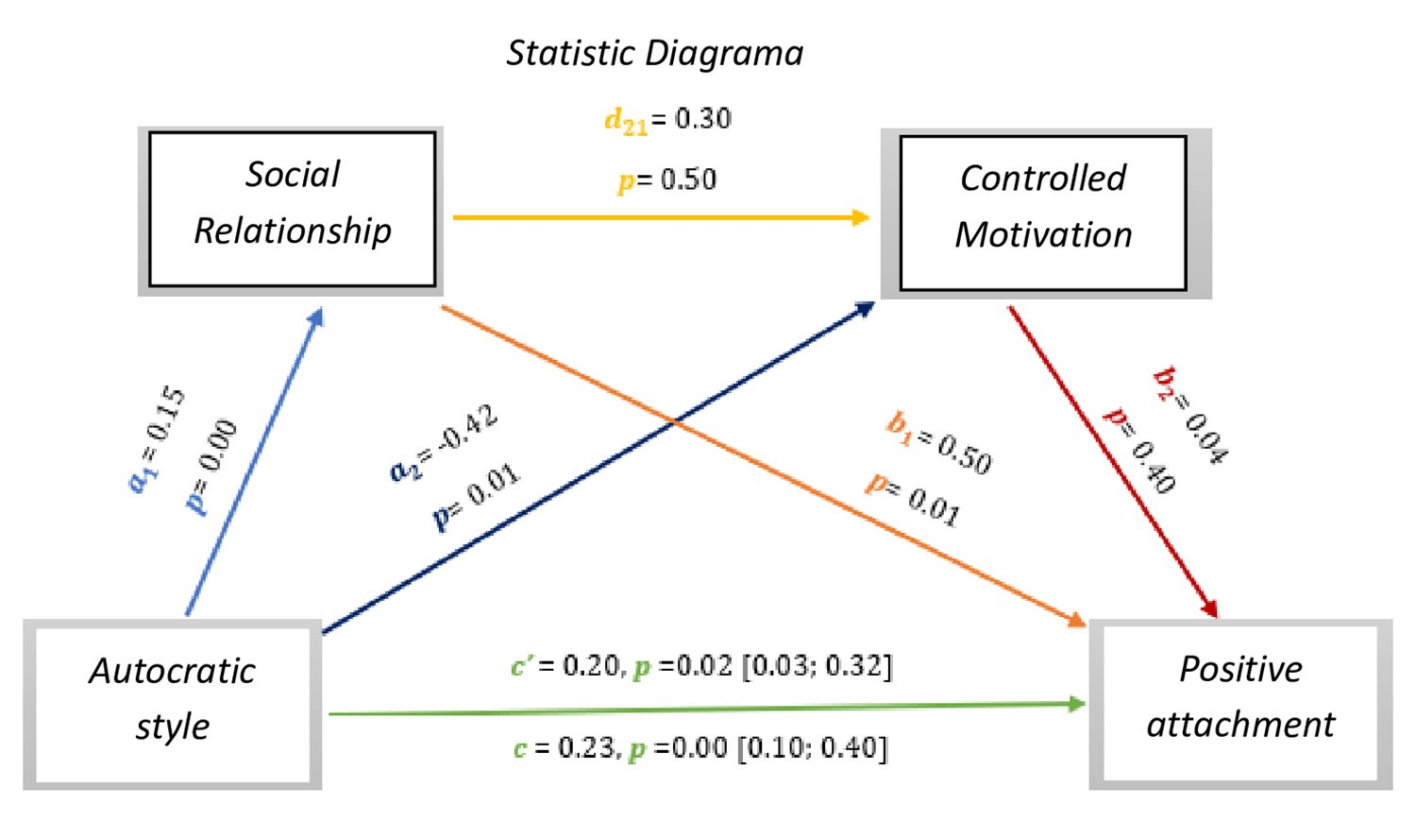
Statistical diagram of the multiple serial model for the relationship between autocratic style (X), positive attachment (Y), relationship (M1) and controlled motivation (M2).

As we can see *in Fig 13,* the results show significant values regarding the direct effect and total effect, *c’* = 0.20, *p* = 0.02, 95% CI [0.03; 0.32], *c* = 0.23, *p* = 0.00, 95% CI [0.10; 0.40], respectively. Although there are no significant effects on the ratio to controlled motivation (0.50) and on controlled motivation for positive affects (0.40).

As for indirect effects, we analyse:

✓ 1° *a*_1_*b*_1_(ind1): X→M1→Y - β = 0.07, SE = 0.03, 95% CI [0.02; 0.20];
✓ 2°: *a*_2_*b*_2_(ind2): X→M2→Y - β = -0.02, SE = 0.02, 95% CI [-0.07; 0.03] e;
✓ 3°: *a*_1_*d*_21_*b*_2_(ind3): X→M1→M2→Y - β = -0.00, SE = 0.00, 95% CI [-0.01; 0.01].

Thus, we found that the autocratic style presents an effect with the positive attachment of athletes with IDD.

**Fig 14:**
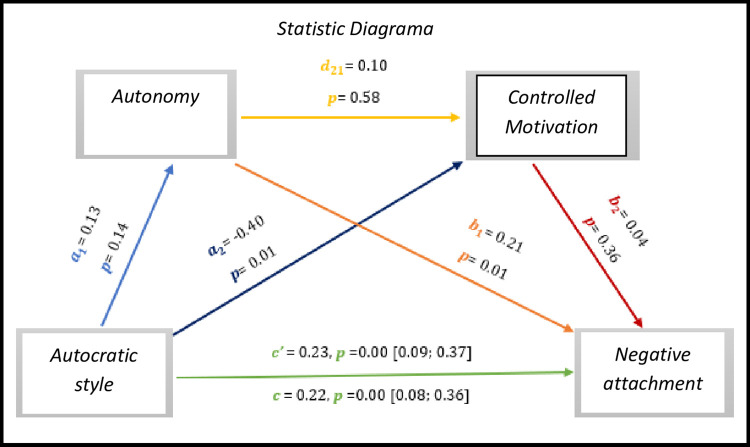
Statistical diagram of the multiple serial model for the relationship between autocratic style (X), positive attachment (Y), autonomy (M1) and controlled motivation (M2).

The results presented in *Fig 14,* identify significant values regarding direct effect and total effect, *c’* = 0.23, *p* = 0.00, 95% CI [0.09; 0.37], *c* = 0.22, *p* = 0.00, 95% CI [0.08; 0.36], respectively. Although there are no significant effects on the autocratic style for autonomy (0.14), on autonomy for controlled motivation (0.58) and on controlled motivation for positive affects (0.36).

As for indirect effects, we analyse:

✓ 1° *a*_1_*b*_1_(ind1): X→M1→Y - β = 0.03, SE = 0.02, 95% CI [-0.01; 0.08];
✓ 2°: *a*_2_*b*_2_(ind2): X→M2→Y - β = -0.02, SE = 0.02, 95% CI [-0.07; 0.03] e;
✓ 3°: *a*_1_*d*_21_*b*_2_(ind3): X→M1→M2→Y - β = 0.00, SE = 0.00, 95% CI [-0.00; 0.00].

We also found that the autocratic style has an effect with the positive attachment of athletes with IDD.

**Fig 15:**
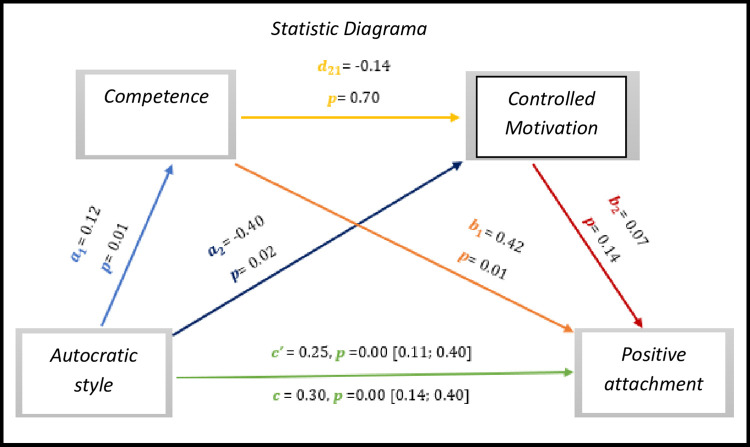
Statistical diagram of the multiple serial model for the relationship between autocratic style (X), positive attachment (Y), competence (M1) and controlled motivation (M2).

Through the analysis of multiple mediation, in *Fig 15,* the results indicate significant values with regard to the total effect (*c* = -0.25, *p* = 0.00, 95% CI [0.11; 0.40]), on the other hand the direct effect is not significant (*c’* = 0.30, *p* = 0.00, 95% CI [0.14; 0.40]).

As for indirect effects, we analyse:

✓ 1° *a*_1_*b*_1_(ind1): X→M1→Y - β = 0.05, SE = 0.03, 95% CI [0.01; 0.11];
✓ 2°: *a*_2_*b*_2_(ind2): X→M2→Y - β = -0.02, SE = 0.02, 95% CI [-0.08; 0.14] e;
✓ 3°: *a*_1_*d*_21_*b*_2_(ind3): X→M1→M2→Y - β = -0.00, SE = 0.00, 95% CI [-0.01; 0.01].

Similarly, we found that the autocratic style presents an effect with the positive attachment of athletes with IDD.

**Fig 16:**
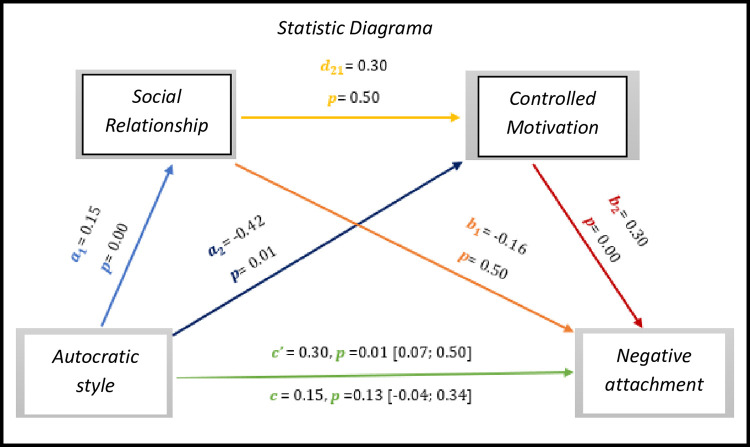
Statistical diagram of the multiple serial model for the relationship between autocratic style (X), negative attachment (Y), relationship (M1) and controlled motivation (M2).

As we can see *in Fig 16,* the results present significant values with regard to the direct effect *c’* = 0.30, *p* = 0.01, 95% CI [0.07; 0.50], on the other hand the total effect is not significant (*c* = -0.15, *p* = 0.13, 95% CI [-0.04; 0.34].

As for indirect effects, we analyse:

✓ 1° *a*_1_*b*_1_(ind1): X→M1→Y - β = -0.02, SE = 0.02, 95% CI [-0.08; 0.02];
✓ 2°: *a*_2_*b*_2_(ind2): X→M2→Y - β = -0.11, SE = 0.05, 95% CI [-0.23; -0.03] e;
✓ 3°: *a*_1_*d*_21_*b*_2_(ind3): X→M1→M2→Y - β = 0.01, SE = 0.02, 95% CI [-0.02; 0.04].

In this sense, the autocratic style presents a direct effect with the negative attachment of athletes with IDD.

**Fig 17:**
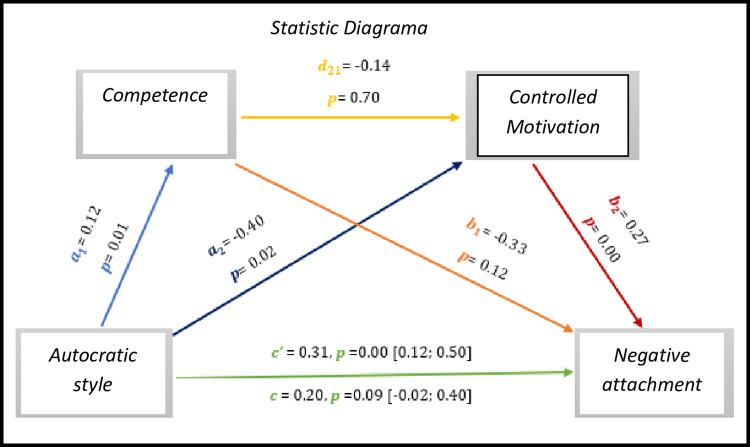
Statistical diagram of the multiple serial model for the relationship between autocratic style (X), negative attachment (Y), autonomy (M1) and controlled motivation (M2).

Based on *Fig 17,* we observed results presenting significant values regarding the direct effect *c’* = 0.30, *p* = 0.01, 95% CI [0.08; 0.45], on the other hand the total effect is not significant (*c* = -0.15, *p* = 0.13, 95% CI [-0.04; 0.40].

As for indirect effects, we analyse:

✓ 1° *a*_1_*b*_1_(ind1): X→M1→Y - β = -0.01, SE = 0.02, 95% CI [-0.04; 0.03];
✓ 2°: *a*_2_*b*_2_(ind2): X→M2→Y - β = -0.10, SE = 0.05, 95% CI [-0.21; -0.02] e;
✓ 3°: *a*_1_*d*_21_*b*_2_(ind3): X→M1→M2→Y - β = 0.00, SE = 0.01, 95% CI [-0.01; 0.02].

Similarly, we found that the autocratic style presents a direct effect with the negative attachment of athletes with IDD.

**Fig 18:**
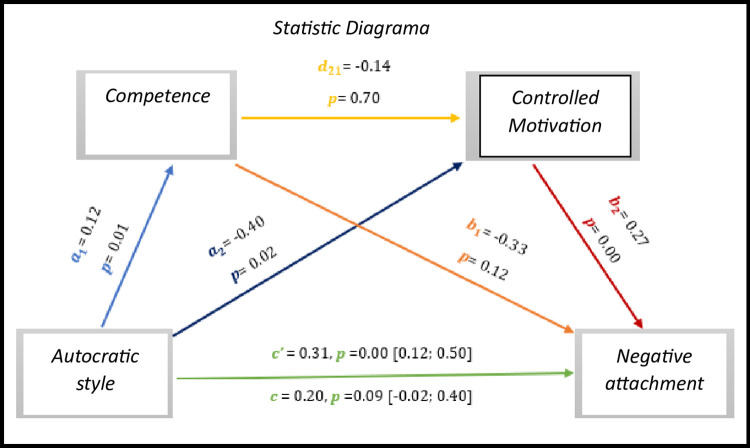
Statistical diagram of the multiple serial model for the relationship between autocratic style (X), negative attachment (Y), competence (M1) and controlled motivation (M2).

The results of *Fig 18,* we observed results present significant values with regard to the direct effect *c’* = 0.31, *p* = 0.00, 95% CI [0.12; 0.50], on the other hand the total effect is not significant (*c* = 0.20, *p* = 0.09, 95% CI [-0.02; 0.40].

As for indirect effects, we analyse:

✓ 1° *a*_1_*b*_1_(ind1): X→M1→Y - β = -0.04, SE = 0.02, 95% CI [-0.10; 0.00];
✓ 2°: *a*_2_*b*_2_(ind2): X→M2→Y - β = -0.10, SE = 0.05, 95% CI [-0.21; -0.02] e;
✓ 3°: *a*_1_*d*_21_*b*_2_(ind3): X→M1→M2→Y - β = -0.00, SE = 0.01, 95% CI [-0.02; 0.02].

Here too, we observed a direct association in the relationship between the autocratic style and negative attachment. In a view, the results show that in large part of all mediation models, in which satisfaction with life, positive attachment and negative attachment are *outcome*, autocratic style has a significant direct effect on increasing basic psychological needs and controlled motivation. However, the democratic style is more consistent in this type of analysis, evidencing with it a greater number of cases with more significant effects and links.

## Discussion

In the sports context, one of the great challenges to be faced is the harmonious relationship between coaches and athletes that, sometimes, the interaction of personal and situational factors is not properly considered, which can interfere in their PA practice, in the Theory of Self-Determination (SDT) and subjective well-being of athletes.

The leadership style of the coach is very important for obtaining good results for his athletes, but little is known about the effects on athletes with IDD, as well as its implications in the practice of PA of these athletes, thus having effects on the functioning and performance of the athlete.

The SDT and subjective well-being have favourable effects on athletes’ performance. A similar study [28] shows that there is a positive influence on autonomous motivation for the practice of PA in Spanish adolescents. On the other hand, in another research [29] there is a demonstration of how the satisfaction of Basic Psychological Needs are good indicators in PA practitioners. Also, other findings [30, 31] found that were effects of the motivational climate on the satisfaction and motivation of basic psychological needs in adolescent athletes.

### Main findings

The objectives of our study were to analyse whether and how basic psychological needs (relationship, autonomy and competence) and autonomous motivation (incorporating into a single index the factors intrinsic regulation and identified regulation) and controlled motivation (incorporating in a single index the factors of introjected and external regulation) mediate the relationship of the variables of the democratic and autocratic leadership style, respectively, with the subjective well-being of athletes with IDD.

Regarding multiple mediation analyses, the main results of our study show that there are significant results for the direct effects of democratic style with satisfaction with life and positive and negative attachment. These findings demonstrate the importance of multicompetent strategies in the context of stimulation of this type of leadership, on the part of the coach, to stimulate the practice of PA in athletes of Adapted Sports with IDD. This has important practical implications for the equity and equal opportunities of the promotion of PA in athletes with this type of condition, evidencing possible strategies on the part of coaches and also the type of multidisciplinary team with whom athletes’ work.

The participants of the present study describe significant values by associating basic psychological needs with autonomous motivation. In another studies [31, 32] adapted sports athletes presented higher values in these variables, when obtaining autonomously motivated behaviours.

The democratic leadership style is a cooperative style, centred on the athlete and oriented to the subject. In the analysis of multiple mediation, our results indicate that the Democratic Style is associated with increased satisfaction with life and positive and negative attachment. Similar results were found [33] when studying Boccia and Basketball athletes in wheelchairs who perceive higher levels of their coaches in democratic behaviour. However, we also researched the style of autocratic leadership, which is a style of command, centred on victory and task-oriented [34]. Here, our results do not indicate that autocratic style is associated with satisfaction with the lives of these athletes, however there is an association with the increase in positive and negative affections. Although we should not forget that athletes with IDD will have, at first, a greater difficulty in terms of autonomy, relationship and competence, because the feeling of well-being [35] or satisfaction with life is closely linked to how the individual is able to cope and absorb the occurrence of episodes of his life, some of these inherent in its own evolution.

Our results are also similar with other findings [36] when studying leadership in sports in a study of the perception of athletes and coaches in the competitive context, where athletes prefer coaches with a more democratic than autocratic style. On the other hand [37] athletes when confronted with their preferences, male athletes prefer autocratic coaches, rather than democratic. More recently [38] when analysing the effects of training and leadership of the Adapted Sports coach in the coach’s planning, obtained results that prove that the coach has predominantly academic training, with democratic leadership style, evidencing together a flexible planning style.

Identifying the role of the coach in motivation, satisfaction with life and positive and negative attachment of athletes, not only provides a new vision to adapt strategies in their behaviour, but also can suggest intervention points and help adapt strategies for the practice of athletes with IDD and, consequently, a better individualized treatment. Given the prevalence of the leadership style of the Adapted Sports coach, this can translate into an increase in the quality of life of its athletes, thus having a considerable impact on SDT and subjective well-being of the same.

One of the strengths of this study was to have shown that the relationship between democratic style and satisfaction with life and positive affections is direct, and that mediators–basic psychological needs and autonomous motivation play a decisive role in this connection. There is also a relationship in this style of leadership with negative attachment, but although negative attachment are not desirable most of the time, in some circumstances it may be the most convenient and functional response, such as fear, which can motivate to avoid danger, and the sadness that can cause renew and originate new action plans after some loss [38, 39]. Another strong aspect is the fact that it includes different internal and external variables to athletes related to the practice of PA, considering aspects of SDT and subjective well-being. The use of this set of variables was important to indicate which factors associated with PA were positively affected by the intervention, as well as allowed the understanding of how the intervention of the coach’s democratic style had an effect on the athlete with IDD. The ways in which the democratic style is associated with satisfaction with life and positive affections are two: in the first, the democratic style determines an increase in satisfaction with life that, in turn, determines basic psychological needs and autonomous motivation; in the second, the democratic style determines an increase in positive affections, which has an impact on increased self-motivation. This analysis also highlights the role of basic psychological needs, because all the paths that link the democratic style to life satisfaction go through it. On the other hand, the autocratic style is not associated with satisfaction with life in these athletes with IDD.

As for the relationship of autocratic style, as we verified earlier, it does not present to have a relationship with satisfaction with life. However, there is a relationship with positive and negative affects, where we find that this relationship is direct and significant. Thus, we cannot say that there is a relationship of this style before subjective well-being because it only shows with positive and negatives affections. Emotional and environmental variations result from complex processes that can be permanent or temporary, where the balance of these situations induces the individual to adapt, promoting well-being [40, 41].

Regarding the most appropriate style for achieving success, the authors suggest that the two dimensions should be adopted simultaneously, since they IDD not find substantial evidence on which of the types of leadership led to greater success [42]. Depending on the characteristics of the situation and the leaders, the same subject can use different leadership styles [43]. On the other hand, reporting to the leadership styles [44], the "ideal" coach would actually have a mix of guiding leadership (with carefully planned strategies and communication skills), humanist (concerned with motivational aspects and a good human resource manager) and democratic (who knows how to listen and accept suggestions and who likes to work as a team). A study [45] with the intention to construct and validate the scales, with the appropriate psychometric properties, one to identify the planning styles and the other to study the decision styles, where it was concluded that the coaches participating in the study had a higher value in the authoritarian and democratic variables. In a compartmentalization of styles [46], the trainer only manifested himself by one of the styles, because neither the autocratic style nor the democratic style were flexible enough to allow the trainer in certain circumstances to be democratic and declaring himself autocratic, and vice versa. The same author states that in relation to the involvement of trainees in the tasks, the behaviour of the trainer influences the way the trainees commit themselves, that is, their willingness to cooperate and carry out the proposed tasks. Recent investigations [47, 48] state that, the training, both formal and informal, conditions the orientation that coaches adopt in the course of their duties. On the other hand, to know the coach reliably, coach profiles were created. These profiles catalogue the coach according to different areas, namely the traditional coach, technological coach, innovative/creative coach, dialogue coach, collaborating coach and critical coach [49], although these profiles should not be considered pure or rigid [50].

Several authors [47, 48, 51–53] revealed through their studies that the coach has an increasingly important role in the training of his athletes, being therefore responsible for a wide range of functions, with a view to the constant and progressive evolution of their performance [54, 55].

This research can be useful for professionals in the fields of sports sciences, psychology, and health, who should reinforce the importance of preserving or improving the motivation and subjective well-being of their athletes in the practice of their modalities and can be a good tool for coaches and for the potentiation of athletes with IDD, not only in their practice but also in their daily life. Also from the evidence on the association between the effects exerted by the coach on self-determined motivation and subjective well-being of the Adapted Sports athlete.

### Practical implications

The importance of practicing physical activity in general population, especially in the population with disabilities, is consensus in the literature, being an essential behavior to promote health, namely the themes addressed in this research (STD) and subjective well-being. However, despite the known benefits, the perception that athletes may have of their coach and the adjacent effects of the coach may influence the type of motivation. The main findings of this work emphasize the importance of promoting PA in people with IDD, especially to increase the self-determined motivation and subjective well-being of athletes with this condition, in order to improve their quality of life and their PA practice. Our data also suggest that, when practicing PA we should not forget the coach-athlete relationship, but rather enhance it so that its positive consequences are productive for the athlete, particularly in their subjective well-being, which can be effective in promoting improvements in the mentioned factors.

### Limitations and future directions

This study has some limitations. First, we cannot make causality inferences due to the cross-sectional design of the study. The use of questionnaires is also limited, and we are studying athletes with IDD, that is, they show some dependence on external caregivers, although there was, in case of need, an assistant during the completion of the questionnaires. Another limitation was the impossibility of achieving a more homogeneous sample in terms of age. On the other hand, we can consider that the extension of the study instruments and the time for their completion was extensive. And finally, the scarcity of information on the subject Adapted Sport as well as the lack of studies applied to the population with IDD.

The limited number of studies included in this study reflects the scant research conducted in individuals with IDD, which determines possible guidelines for future studies. Thus, we should study this theme with a representative sample, as well as it would be pertinent to carry out longitudinal or experimental investigations. In addition to studying the implications of this training of the coach in the athlete, itis also important in the in-depth determination of the theme in athletes with IDD. In addition, it would be pertinent to verify the effects of the coach’s leadership style on self-determined motivation and subjective well-being of the athlete with IDD confronting the different modalities, as well as considering representative samples, so it would also be pertinent to conduct longitudinal or experimental research for a more in-depth understanding of the theme.

Through the experience gained throughout this investigation, we suggest some proposals that we consider interesting for future investigations: a) Replicate these studies, but with more representative samples; b) cover athletes with other types of disabilities; c) Using qualitative research as a complement to quantitative, in order to identify personal or particular aspects; d) In addition to measuring the athlete’s perception, it may also be relevant, identify the coach’s self-perception of himself, and also, the coach’s preferences athlete over coach; e) Evaluate the motivational climates promoted by coaches and what effect exerts on the variables of self-determination and consequences studied.

## Conclusions

The relationship between the democratic style and the satisfaction with life, the positive attachment and the negative affects reach a direct effect, and the mediating variables, basic psychological needs and autonomous motivation, assume a decisive role in this connection, while the autocratic style does not produce a direct effect with satisfaction with life, evidencing only relationship with positive and negative attachment. These findings demonstrate the importance in the creation of multicompetent strategies in the context of the stimulation of the type of Democratic leadership, on the part of the coach, to stimulate the practice of PA in athletes of Adapted Sports with IDD, which consequently will have important practical implications for the equity and equal opportunities of the promotion of PA in athletes with this type of condition, evidencing possible strategies on the part of coaches and also the type of multidisciplinary team with whom athletes work.

In conclusion, our results hurt the existence of a mediation effect between those of basic psychological needs and the autonomous motivation, exercised between the democratic leadership profile of the coach and the subjective well-being of the athlete. Our results also reinforce the importance of promoting a relationship in the style of leadership on the part of the coach in their athletes, thus activating a subjective well-being higher than the part of the athletes.
